# Bioefficacy of an Oil-Emulsion Formulation of Entomopathogenic Fungus, *Metarhizium anisopliae* against Adult Red Palm Weevil, *Rhynchophorus ferrugineus*

**DOI:** 10.3390/insects14050482

**Published:** 2023-05-20

**Authors:** Cheong Jia Lei, Raja Hasya Ilyana Raja Ahmad, Najihah Abdul Halim, Norhayu Asib, Azlina Zakaria, Wahizatul Afzan Azmi

**Affiliations:** 1 Faculty of Science and Marine Environment, Universiti Malaysia Terengganu, Kuala Nerus 21030, Terengganu, Malaysia; cheongjialei@gmail.com (C.J.L.); hasyailyana106@gmail.com (R.H.I.R.A.); najihahhalim_94@yahoo.com (N.A.H.); 2 Department of Plant Protection, Faculty of Agriculture, Universiti Putra Malaysia, Serdang 43400, Selangor, Malaysia; norhayuasib@upm.edu.my; 3 Sime Darby Research Sdn. Bhd., KM10, Jalan Banting-Kelanang, P.O. Box 207, Banting 42700, Selangor, Malaysia; azlina.zakaria@simedarbyplantation.com

**Keywords:** *Metarhizium anisopliae*, entomopathogenic fungus, red palm weevil, oil-emulsion formulation

## Abstract

**Simple Summary:**

This study aimed to explore the effectiveness of a new microbial formulation that utilizes the entomopathogenic fungus, *Metarhizium anisopliae*, in controlling the red palm weevil (RPW), *Rhynchophorus ferrugineus*, which is the most destructive insect pest of major cultivated palms. Previously, we published the development of an oil-emulsion formulation system to enhance the biological performance of this particular fungal isolate. The system improved the stability, replicability, shelf life, and resistance to heat stress and UV irradiation. The current results reveal that the formulation has improved fungal bioefficacy and pathogenicity through direct and indirect contact bioassays. The lethal infection of formulated conidia against RPW was more effective and dispersed better than the aqueous suspension of non-formulated conidia. The spread of disease can be observed from a treated individual to a healthy RPW upon contact, which explains the disease-spreading ability of formulated conidia. Additionally, the infections were confirmed through both morphological analysis and DNA sequencing. The results showed that the RPWs were exclusively infected by *M. anisopliae* throughout the experiments. Therefore, this oil-emulsion formulated conidia could be incorporated into current integrated pest management programs to achieve more promising biocontrol outcomes in the long-term application of sustainable agricultural practices.

**Abstract:**

The red palm weevil (RPW), *Rhynchophorus ferrugineus*, poses a severe threat to agro-industrial crops, particularly major cultivated palm species. Infestations result in economic losses due to reduced fruit quality and yield. The entomopathogenic fungus, *Metarhizium anisopliae*, has shown promise as a potential biocontrol agent against the RPW. However, the use of an emulsion formulation of *M. anisopliae* for managing this serious insect pest has yet to be fully explored. The oil-emulsion formulation containing this entomopathogen may enhance the conidia’s stability, prolong its lifetime, and reduce the impact of heat stress or UV irradiation on the fungus. Therefore, this study aimed to investigate the bioefficacy of a new oil-in-glycerol emulsion formulation on mycoinsecticidal activity against RPW adults by direct and indirect bioassays. Results showed that conidia concentration was directly proportional to the RPW mortality percentage. The LT_50_ of 8.183 days was achieved by the conidial formulation against RPW, with a significantly lower LC_50_ (1.910 × 10^5^ conidia mL^−1^) compared to the aqueous conidia suspension (LT_50_ = 8.716 days; LC_50_ = 7.671 × 10^5^ conidia mL^−1^). Indirect bioassays revealed that the oil-in-glycerol emulsion had a disease-spreading ability that resulted in up to 56.67% RPW mortality. A zero E-value reading indicates that the DNA sequence being studied is highly similar to that of the fungal species *M. anisopliae*, which has been identified in the NCBI database. Although the new emulsion formulation has improved the efficacy and pathogenicity of *M. anisopliae* in vitro, it is important to also consider the fungal pathogen’s compatibility with other agricultural practices to prevent any loss of control efficiency in the actual usage environment.

## 1. Introduction

The red palm weevil (RPW), *Rhynchophorus ferrugineus* (Coleoptera: Dryopthoridae), is a significant insect pest that affects many palm species. This weevil has severely threatened palm species worldwide, emanating from Southeastern Asia as well as South and Melanesian countries, and has widely spread among commercial palms, such as *Phoenix dactylifera*, *Cocos nucifera*, *Elaeis guineensis*, and *Metroxylon sagu*, due to the insect polyphagous nature [1]. Poor biosecurity practices in the global trading of infested palms to extended barriers have accounted for nearly all of its rapid colonization, where it has successfully invaded parts of East Asia, the Middle East, the Mediterranean Basin, and the Caribbean [1,2].

Of late, RPW infestation in Malaysia has intensified due to the unmonitored trade of infected seedlings or mature palms, which has become a global issue [2,3]. The situation is doubly devastating due to the concealed nature of the larvae as they burrow into the palm’s trunk. This destructive action weakens and eventually kills the host plant. At the same time, planters regularly apply insecticides and extend the implementation period primarily to effectively manage the established population of RPW [4,5].

The natural tendency of coleopteran pests, particularly RPW, to evolve insecticide resistance in palm cultivated fields is still unclear. As a result, biocontrol using bio-inoculants could be a sustainable approach to managing insect pests, given the environmental burden and health risks associated with insecticides in agricultural fields. *Metarhizium anisopliae* (Hypocreales: Clavicipitaceae) is a promising entomopathogen of RPW. Biological control using *M. anisopliae* does not solely rely on highly infectious propagules. However, conidia gradually decrease in viability over time due to prolonged storage and unconducive environmental conditions [6,7]. This can result in a lower mortality rate for insect pests after direct or indirect contact infection. Therefore, the formulation and delivery system should aim to stabilize and enhance the storability of the product while also facilitating effective delivery to the weevils.

Conidial formulations can take various forms, including emulsions, emulsifiable concentrates, suspension concentrates, wettable powders, and granules [8]. Any alternative formulation would be a desirable alternative to replace the current biocontrol strategy of using *M. anisopliae* spore suspension in terms of shelf life and ease of application. Many technically viable products of *M. anisopliae* are still in use in both local and global agricultural pesticide markets. For instance, Ory-X^®^ (FELDA Agricultural Services Pte. Ltd., Kuala Lumpur, Malaysia) produces a mixture of dry spore powder and emulsifier that can be mixed easily with water to form a sprayable suspension. This suspension is used to target the rhinoceros beetle (*Oryctes rhinoceros*) by spraying it onto rotting palm trunks or fronds [8]. In the commercial development of *M. anisopliae* bioformulations, production is not economically cost-effective unless the formulation is viable, virulent, and stable for a prolonged period of time.

A laboratory study has demonstrated the virulence of *M. anisopliae* (strain Met-Gra4) against RPW [9]. However, the current biocontrol technique that uses dry-harvested conidia powder has revealed a significant decline in conidial viability over time [10]. Cheong et al. [11] developed a formulation system to enhance the biological performance of this particular fungal isolate. The system aims to achieve a high success rate of conidial adherence to the RPW cuticle upon germination while minimizing environmental damage to the viable conidia. Until now, no registered fungal-based insecticide has been used to control RPW in Malaysia. Despite the availability of more actionable research, the market demand for a viable alternative bioformulation against RPW still needs to be met. Thus, it is necessary to verify if the oil-emulsion formulation of the fungal strain corresponds to the target species and to develop an appropriate formulation based on biological control strategies.

Therefore, this study aimed to evaluate the in vitro pathogenicity of oil-emulsion formulated and non-formulated *M. anisopliae* conidia against RPW adults using direct and indirect contact bioassays. The results were then further verified through morphological and molecular identification of the fungal infection.

## 2. Materials and Methods

### 2.1. Source of Red Palm Weevils (RPW)

The RPW adults were collected using pheromone traps baited with a mixture of 90% 4-methyl-5-nonanol and 10% 4-methyl-5-nonanone and kairomone-releasing fermented pineapples. These traps were positioned under the shade of coconut palms around the Universiti Malaysia Terengganu (UMT) campus (coordinated at 5°19′49.0008″ N, 103°8′30.6564″ E). RPW adults were reared in the laboratory using plastic containers (dimensions of 45 × 12 × 17 cm), which had a hole on the top and sugarcane as a food source. For mating procedures, one male and one female RPW were confined together in a wooden rearing box (dimensions of 18 × 18 × 25 cm) under ambient temperature conditions of 24 ± 2 °C, 70 ± 5% RH, and a photoperiod of 12:12 (L:D) h. Food substrates, including sugarcane and decayed substrates, were provided for the adults and replaced twice weekly. The rearing boxes were emptied on a daily basis, and the medium was examined for eggs. The eggs were placed individually in small circular plastic containers, and hatching young larvae were then reared individually in 2.5 L containers, each filled halfway with a 1:1 mixture of sugarcane and sawdust. Food media were replaced every 2–3 weeks until the emergence of adults. Upon emergence, the adults were fed as previously described.

### 2.2. Pathogenicity Test of M. anisopliae

Before conducting the pathogenicity test, healthy male and female RPW with complete body parts, free of mites, and measuring 3.0–3.5 cm in length were selected. Selected male and female RPW were sterilized by dipping them in 70% alcohol for no more than 15 s, followed by three simultaneous dips in sterile distilled water.

The pure culture of *M. anisopliae* strain Met-Gra4 was obtained from Grace Lee (PhD, UMT). The conidia of Met-Gra4 were freshly grown, mass-produced through solid-substrate fermentation, and subsequently dry-harvested using a sieve shaker. The drying temperature for post-fermentation solid substrate was maintained at room temperature (25 °C ± 3 °C). The spore viability was assessed before using them in experiments. Only *M. anisopliae* with a high viability of over 70% were selected and used for subsquent experiments.

A stock of an oil-in-glycerol emulsion formulation containing *M. anisopliae* strain Met-Gra4 was prepared following the method of Cheong et al. [11] with minor modifications. The conidia were prepared in two forms: oil-emulsion formulated and non-formulated (dry-harvested conidia suspended in 0.05% aqueous Tween 80). The concentrations of both types were adjusted to a concentration of 1 × 10^3^, 10^5^, 10^7^, and 10^9^ conidia mL^−1^ for the treatments. All treatments were prepared at least two weeks before being used in the experiments.

A total of 720 healthy individuals of the RPW were used in this study. The disinfected RPWs were sprayed with the prepared conidial suspensions for around 60 s (~1 mL) to ensure that the spores were evenly distributed on the surface of the weevils’ bodies. The inoculated RPWs were kept in containers at a temperature of 25 ± 1 °C and a photoperiod of 12 h (L)/12 h (D). The lid of each container had small holes punctured in it. Three replicates were used for each treatment, with 10 RPWs per container (560 mm in length × 270 mm in width × 360 mm in height).

Control treatments involved RPW treated with a diluted blank formulation and untreated RPW. Commercial mycoinsecticide (Ory-X, FELDA Agricultural Services Pte. Ltd., Kuala Lumpur, Malaysia) and pesticide (Cypermethrin, Compesti Pte. Ltd., Puchong, Malaysia) were used as positive controls. The commercial pesticide solutions were prepared according to the manufacturer’s protocol. The concentration prepared for Ory-X was 1 × 10^7^ conidia mL^−1^, whereas the concentration for cypermethrin was 110 ppm. Similar amounts of solutions were applied to RPW using the same methods and replicates.

Pathogenicity tests included herein comprise both direct and indirect bioassays. All RPW individuals were inoculated with the aforementioned concentrations for direct contact bioassay. On the other hand, indirect contact involved only one inoculated RPW in each treatment to simulate the spread of the disease among the healthy group of adult RPW in the field. Fungal-induced behavioural changes of RPW in each treatment were assessed daily for two weeks after each treatment.

### 2.3. Morphological Identification of M. anisopliae

*Metarhizium anisopliae* pure culture was obtained by dry-harvesting conidia through solid-substrate fermentation, and infected thoracic or cephalic muscle tissues of RPW cadavers (treated with 1 × 10^6^ conidia mL^−1^) were grown on PDA medium. One-week-old fungal pure cultures were used for morphological identification. The methodology for morphological identification was based on Ishak et al. [12], with some modifications. The glass slides and coverslips were prepared by spraying them with 70% ethanol and allowing them to dry. A square with a diameter of 5 × 5 mm was cut from the PDA using a sterile scalpel blade. The agar pieces were then placed on a glass slide and covered with a coverslip. The characteristics of *M. anisopliae* colonies were studied macroscopically by observing their colour, shape, surface, and hyphae. Microscopic observations were made on the hyphae, conidiophores, and conidia using a compound microscope at 100× magnification [13,14].

### 2.4. Molecular Identification of M. anisopliae

For fungal DNA extraction, one-week fungal pure cultures from the treatments mentioned above were used. The mass of fungal mycelia was scraped out (without agar medium) using a fine spatula, and 0.1 g of the mass was transferred into a sterile porcelain mortar and ground with liquid nitrogen using a pestle. Fungal genomic DNA was extracted from lyophilised fungal masses using the Nucleospin^®^ DNA extraction kit (MACHEREY-NAGEL™).

The extracted DNA was assessed for both purity and quantity using a NanoDrop One spectrophotometer (Thermo Scientific™, Wilmington, DE, USA). The ITS region of the ribosomal RNA (rRNA) gene in fungi was amplified using universal primers ITS1 (5′ TCC GTA GGT GAA CCT GCG G 3′) and ITS 4 (5′ TCC TCC GCT TAT TGA TAT GC 3′). The PCR amplification was carried out in a final volume of 25 µL, which included approximately 50 ng of DNA template, 12.5 µL of GoTaq^®^ Green Master Mix (Promega, Madison, WI, USA) as a substitute for Taq DNA polymerase, dNTPs, MgCl2, and reaction buffer, 0.5 µL of each amplification primer (10 µM), and nuclease-free water. Nuclease-free water was used to replace the template DNA in the negative control. The PCR reactions were amplified using a thermocycler machine (T100 Thermal Cycler, Bio-Rad, Hercules, CA, USA) with an initial denaturation at 95 °C for 3 min, followed by 30 cycles at 95 °C for 30 s, 51 °C for 1 min, and 72 °C for 1 min, and a final extension was performed at 72 °C for 5 min.

The size and quality of the aliquot PCR products (1 µL) were determined through gel electrophoresis. Approximately 1000 base pairs (bp) of PCR products were separated electrophoretically on 1% agarose gels during the second round of amplification. The gels were then stained with Diamond™ Nucleic Acid Dye (Promega, Madison, WI, USA) and viewed under UV illumination with a maximum excitation of 495 nm. The gel was run for 60 min at 100 V. The size of the fragments was determined by comparing them to a 1 kb DNA ladder (Promega, Madison, WI, USA) and analysed using the Gel Doc XR Gel Documentation System. The size of the PCR product is estimated to be 578 base pairs. The amplicons were then sent to 1st BASE Laboratories Pte. Ltd. (Shah Alam, Malaysia) for sequencing analysis. The ITS sequences obtained from *M. anisopliae* samples were compared to the *M. anisopliae* sequence database using the Basic Local Alignment Search Tool (BLAST) in GenBank. The nucleotide similarity between sequences must be greater than 99% to confirm that the samples are true *M. anisopliae*.

### 2.5. Statistical Analysis

All analyses were conducted using IBM SPSS Statistics 25 software at a significance level of α = 0.05. Levene’s test was used to assess the homgeneity of variances for a variable computed across two or more groups. Lethal concentrations (LC_50_ and LC_90_) for the host susceptibility test were determined using the EPA Probit Analysis program version 1.5. The LT_50_ for certain treatments was calculated using linear regression in Microsoft Excel. The Student-Newman-Keuls (SNK) test was then conducted to determine any significant differences between the treatments. R^2^ was used to assess the strength of the relationship between the LT_50_ values of different treatments in each bioassay. The infectivity of the conidial formulation was adjusted by subtracting the natural mortality observed in the control treatment (0–5%) using Schneider Orelli’s formula [11]:

$$Corrected\;efficacy\;\%=\frac{\left(treated\;mortality\;\%)-(untreated\;mortality\;\%\right)}{100-(untreated\;mortality\;\%)}\times 100$$

## 3. Results

### 3.1. Pathogenicity Tests

The efficacy of oil-emulsion formulated conidia, and a non-formulated control (dry harvested conidia in a 0.05% aqueous Tween 80 suspension) was tested on adult RPW based on four concentrations, adjusted to 1 × 10^3^, 1 × 10^5^, 1 × 10^7^, and 1 × 10^9^ conidia mL^−1^ (Table 1). Zero mortality was observed for the negative control treatments, which confirmed the entomopathogenic effect of *M. anisopliae* conidia on RPW. Cypermethrin, which served as the positive control, showed a higher mortality rate (LT_50_ = 5.138 days) compared to the other treatments.

Overall, the higher concentration of conidia resulted in faster mortality rates. Results showed that higher spore concentrations resulted in increased mortality rates due to a greater probability of infection. Mortality began as early as the second day for the oil-emulsion formulated conidia treatment (1 × 10^9^ conidia mL^−1^), and reached 100% after 14 days. For the treatment with non-formulated conidia (1 × 10^9^ conidia mL^−1^), mortality began on the third day and reached 100% on the 14th day.

Levene’s test for equality of error variances showed significant evidence to reject the assumption of equal variances (*F*_0.05,111,224_ = 2.829, *p* = 0.000). Two-way ANOVA with SNK post hoc test indicated a significant difference in emulsion at 1 × 10^9^ conidia mL^−1^ and cypermethrin among all treatments (*p* < 0.05). An R^2^ value above 0.8 collectively indicated a better fit to the linear regression model for all treatments. The LT_50_ scores for emulsion formulated conidia decreased as concentration decreased, ranging from 8.183 to 49.865. In contrast, the LT_50_ scores for non-formulated conidia against RPW ranged from 8.716 to 70.721 days after inoculation. However, there was no significant difference observed between the emulsion formulated and non-formulated conidia at a concentration of 1 × 10^9^ conidia mL^−1^. In the negative control treatment, no infection was observed on RPW when treated with Ory-X in a direct contact bioassay.

The pathogenicity tests conducted on RPWs showed a significant difference among all four conidial concentrations that were tested; oil-emulsion formulated conidia [χ^2^(2, *N* = 30) = 1.021, *p* < 0.05] and non-formulated conidia [χ^2^(2, *N* = 30) = 4.070, *p* < 0.05] (data not shown). The estimated LC_50_ and LC_90_ values reveal that the emulsion formulated conidia require a lower concentration to eliminate 90% of the RPW population at LC_90_ = 3.893 × 10^6^ conidia mL^−1^, compared to non-formulated conidia at LC_90_ = 2.638 × 10^8^ conidia mL^−1^ (Table 2).

The use of oil-emulsion formulated conidia from a single infected RPW individual in an indirect contact bioassay showed potential for bioformulation in disease transmission. Negative controls using an emulsion blank and a 0.05% Tween 80 aqueous solution showed zero RPW mortality. A positive control using a non-ecofriendly chemical pesticide (cypermethrin) induced 100% mortality, while Ory-X (a commercial mycoinsecticide) caused no RPW mortality. As the concentration of emulsion formulated conidia increased, the infection rate also increased, ranging from 10% to 56.67%. In contrast, non-formulated conidia could not spread fungal-induced diseases among healthy RPW individuals in any of the treatments. The mortality score for this treatment was determined by infected RPW individuals in all replicates.

As the concentration of oil-emulsion formulated conidia increased, the LT_50_ of the treatment decreased, ranging from 50.679 days to 12.475 days (Table 3). There was a statistically significant difference between the three treatments at different concentrations, as determined by the White test for heteroskedasticity [2(8) = 169.562; *p* < 0.001]. A two-way ANOVA with the SNK post hoc test indicated a significant difference in the LT_50_ observed for the emulsion at 1 × 10^9^ conidia mL^−1^ and cypermethrin compared to all treatments (*p* < 0.001). There is no significant difference in the LT_50_ values between non-formulated conidia treatments and emulsion formulated conidia treatments at 1 × 10^3^ conidia mL^−1^. The R^2^ value above 0.8 collectively indicates a better fit for the linear regression model across all treatments.

**Figure 1:**
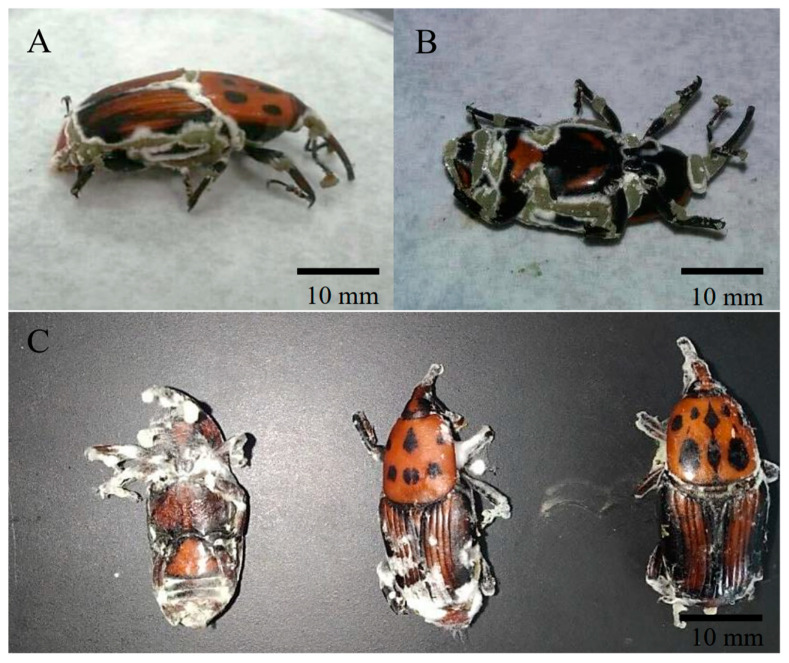
Cadavers of infected RPW treated with oil-emulsion formulated conidia (**A**,**C**) and non-formulated conidia (**B**).

RPWs that were treated with oil-emulsion formulated conidia or non-formulated conidia appeared hardened and reddish brown or retained their body coloration. White hyphae of *M. anisopliae* grew out of the cadaver surface and turned green after few days, regardless of the treatment concentration (Figure 1). A delay in hyphal growth was observed on RPWs treated with oil-emulsion formulated conidia, likely due to the slight oily surface of the treatment. Other saprophytic fungi other than *M. anisopliae* can be found on RPWs that have been treated with emulsion formulated conidia. Unidentified fungi with white filamentous hyphae were grown on hardened RPW cadavers. The infection of *M. anisopliae* was confirmed through molecular analysis, which showed that all RPW were infected solely by the conidia of *M. anisopliae*.

### 3.2. Morphological and Molecular Verification

**Figure 2:**
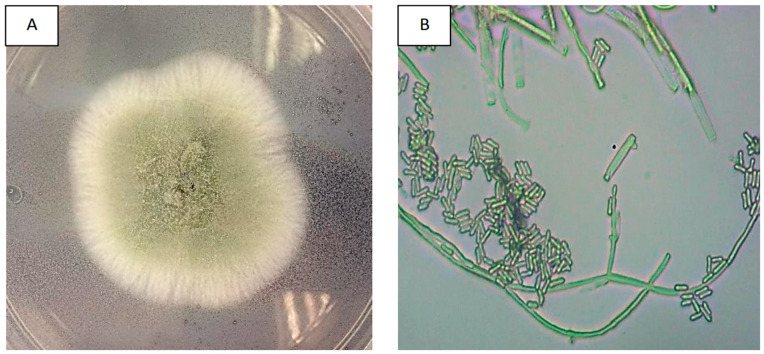
Macroscopic and microscopic photographs of *M. anisopliae* strains; front view of *M. anisopliae* colonies in PDA medium (**A**); and microphotographs of *M. anisopliae* conidial germination at 100× magnification (**B**).

All fungal isolates from pure cultures, dry-harvested conidia, and infected tissues of RPW cadavers showed similar morphological characteristics. After a week of incubation, they formed white colonies that developed into compact green conidiophore clusters, which are typical macroscopic characteristics of *M. anisopliae* (Figure 2A). The microscopic observations showed septate mycelium and branched conidiophores resembling candelabras, with two or three branches in each septum. Their conidiogenous cells were cylindrical with rounded edges and displayed the olive-green colour characteristic of *M. anisopliae* (Figure 2B).

**Figure 3:**
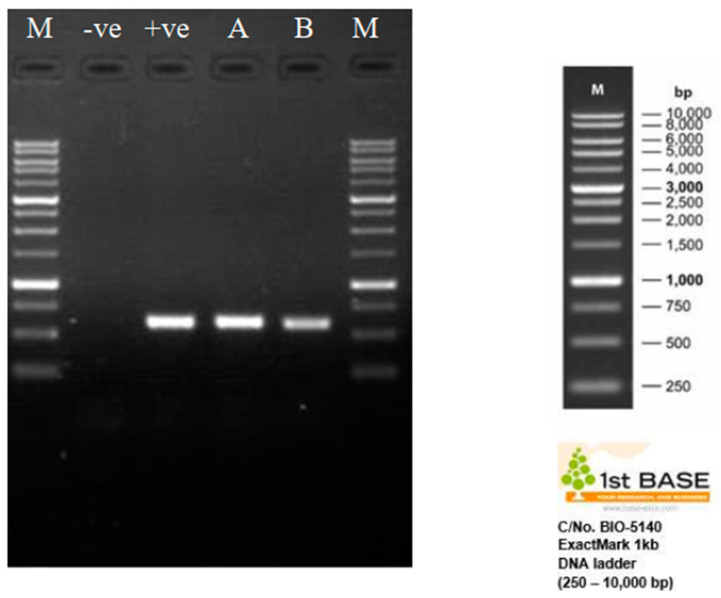
The PCR product for the fungal isolate. M = 1 kb DNA ladder; −ve = PCR non-template control; +ve = DNA extracted from *M. anisopliae* pure culture; A = fungal DNA extracted from RPW treated with a conidia suspension (1 × 10^7^ conidia mL^−1^); B = fungal DNA extracted from RPW treated with emulsion formulated conidia.

Results of the sequence BLAST hits against the NCBI nucleotide (nr/nt) database showed that 99.64–100% homolog to *M. anisopliae*, with an expected value (E) of zero. Significant matches were found under the following accession numbers when randomly browsing a database related to subject of interest: KX806656.1, MH512951.1, KX809518.1, MT448733.1, MG917658.1, KX809519.1, FJ545279.1, HM055447.1, AY646393.1, and MH104861.1. Therefore, all of the isolates remained consistent throughout the bioassay, making it a suitable starting point for entomopathogenic studies aimed at developing durable blast-resistant varieties. Based on the ExactMark 1 kb DNA marker (250–10,000 bp), the resultant bands were observed on a 1% TAE agarose gel between 500 and 750 base pairs (Figure 3).

## 4. Discussion

Underlying attenuation mechanisms are often overlooked, despite their impact on the fungal bioefficacy and commercial viability of *M. anisopliae*. Importantly, the adhesiveness of conidial to the host cuticle surface is a key factor in fungal pathogenicity, which, in turn, makes insect pest mortality concentration-dependent [15,16]. Poor conidial adhesion reduces the rate of disease initiation in treated insect hosts, even if a high number of viable conidia are applied. The oil-in-glycerol formulation described here facilitates the adhesion of conidia to the weevil by altering the fungal hydrophobicity. This alteration triggers germination and promotes fungal penetration by disrupting the waxy layer of the insect cuticle [11]. Indeed, oil-assisted conidial adhesion is essential for efficient and indirect biocontrol of insect pests, primarily coleopterans [7].

The present study showed that formulating *M. anisopliae* conidia in an oil-in-glycerol emulsion increased the pathogenic potential of the fungus. The most effective results were obtained when the fungal conidia were included in an emulsion. At a concentration above 1 × 10^7^ conidia mL^−1^, the mortality percentages of RPW adults were close to 100% within two weeks. Entomopathogenic fungi may desiccate at low relative humidity and fail in conidiogenesis. However, the RPW cadaver microenvironment may regulate conidial germination and toxic metabolites production [10,17]. The mechanisms by which some EPF strains are more pathogenic under drier conditions are unclear. Nevertheless, their high biological efficacy is associated with increased water activity in the insect host. The conidiogenesis process of *M. anisopliae* was a dynamic and depends not only on a conducive humidity level (70 ± 5% RH), sporadically or incessantly throughout the assays, but also on the cadaver’s potential to absorb water [18].

Oil-in-glycerol emulsion promotes the dispersion and adhesion of conidia on the cuticle of insect hosts [19,20]. It also protects conidia from desiccation in low humidity environments [21], and minimises the adverse effects of abiotic environmental factors such as heat stress [22] and ultraviolet radiation [23]. Furthermore, utilizing an emulsion formulation for *M. anisopliae* conidia offers distinct advantages in terms of improving shelf-life, achieving high mortality rates in insect hosts, and protecting conidia from imbibitional damage during dilution. Oil-emulsion formulation strategies can enhance the effectiveness and pathogenicity of fungi [11]. As demonstrated in the present study, infected individuals were capable of spreading the infection to other healthy individuals while in captivity.

Although the present study did not focus on the expression of fungal enzymes, the information mentioned below may help explain the host-fungi interaction within the emulsion system. According to Shah et al. [24], a spore-bound enzyme (protease, Pr1) is a significant insecticidal factor that is independent of hydrophobicity and electrostatic force. However, the entomopathogenic mechanism varies depending on the strain. These cuticle-degrading enzymes are crucial for the initial adhesion and penetration of the fungus, as they also supply and provide nutritional resources to the fungal germ tubes [17]. Shah et al. [24] proposed that de-repression of enzyme Pr1 could be induced and generated through starvation stress, which may occur in conidial stock emulsion after prolonged storage. Increased levels of this enzyme have been shown to enhance fungal entomopathogenicity. In contrast, Pr1 levels declined when it was successively subcultured on nutrient-rich media, such as potato dextrose agar. Fungal pathogenicity greatly depends on the coordinated interaction between various virulent determinants. Although the spore-bound enzyme is crucial for adhesion, prolonged storage of dry conidia at room temperature may lead to excessive desiccation, which can debilitate conidial reactivation from dormancy [25] and be highly susceptible to imbibitional damage [26].

The current study showed enhanced conidial infectivity in a glycerol blend, even under suboptimal ambient relative humidity conditions (below 65%). Since the conidia may adhere for a significant period until environmental conditions, such as humidity, and become favourable for enzyme secretion and penetration of the host cuticle, the hygroscopic nature of glycerol plays an essential role in absorbing ambient moisture and creating a conducive microenvironment on the insect cuticle. Additionally, the previous studies using non-formulated fungal strain MetGra4 (>80% viable conidia) have confirmed the increased efficacy of emulsion formulated conidia, wherein Fong et al. [27] and Insyirah et al. [12] reported the use of 5.00 × 10^7^ conidia mL^−1^ and 5.40 × 10^6^ conidia mL^−1^ aqueous suspension, which achieved a LT_50_ of 7.33 days and 8.66 days, respectively.

The formulated conidia in the present study recorded an LC_50_ of 1.91 × 10^5^ conidia mL^−1^ and an LT_50_ of 8.18 days, which is significantly lower than that of the non-formulated conidia suspension reported in previous studies. When bioassayed against adults of *R. ferrugenius*, the mortality percentage and LT_50_ considerably favoured formulated conidia compared to conidial suspension in water, as the low wetting angle between oil and lipophilic surfaces further improves epicuticle disruption and facilitates cuticle adhesion. Insect mortality in the indirect bioassay also confirmed the synergistic effect of the emulsion and fungal properties. Therefore, the emulsion formulation of *M. anisopliae* conidia is likely to create a conducive microenvironment on insect cuticles by providing adequate free water for conidial germination even under low humidity conditions, which in turn helps to spread the disease over time.

## 5. Conclusions

The results of the pathogenicity test for oil-emulsion formulated conidia showed a high rate of lethal infection of *M.anisopliae* against RPW. Emulsion formulated conidia disperse better than those in an aqueous suspension with a surfactant, ensuring a higher chance of conidia adhering to insect hosts, and leading to a more balanced and repeatable application, which explains the disease spreading ability of formulated conidia. Additionally, the infections were verified through morphological characterization and DNA sequencing, of which the RPWs were exclusively infected by *M. anisopliae* throughout the experiments. This new fungal formulation will serve as a more environmentally friendly alternative to reduce the use of harmful chemical pesticides in current integrated pest management programmes, especially in Southeast Asia, where important economic yield and the production of palm species, such as sago palm, coconut palm, and oil palm, can be secured.

## Figures and Tables

**Table 1:** Direct contact bioassay using oil-emulsion formulated conidia and non-formulated conidia at different concentrations.

Direct Contact Bioassay				
Treatment	95% Confidence Level			
	LT_50_ (Days)	Lower Bound	Upper Bound	R^2^
Oil-emulsion formulated conidia				
1 × 10^3^ conidia mL^−1^	49.865 ^ab^	5.165	21.025	0.837
1 × 10^5^ conidia mL^−1^	18.0359 ^bc^	13.499	29.358	0.959
1 × 10^7^ conidia mL^−1^	8.951 ^de^	35.404	51.263	0.959
1 × 10^9^ conidia mL^−1^	8.183 ^c^	39.689	55.549	0.988
Non-formulated conidia suspension				
1 × 10^3^ conidia mL^−1^	70.721 ^a^	−2.454	13.406	0.915
1 × 10^5^ conidia mL^−1^	27.273 ^ab^	5.880	21.739	0.926
1 × 10^7^ conidia mL^−1^	11.496 ^cd^	23.499	39.358	0.874
1 × 10^9^ conidia mL^−1^	8.716 ^de^	35.880	51.739	0.949
Positive controls				
Cypermethrin (pesticides)	5.138 ^f^	63.909	75.116	0.898
Ory-X (mycoinsecticides)	0	0	0	0
Negative control				
0.05% Tween 80	0	0	0	0
Emulsion blank				

Note: According to the Student-Newman-Keuls post hoc test, the LT_50_ values followed by different letters are significantly different at *p* = 0.05 (n = 30). ‘0’ represents zero mortality.

**Table 2:** The estimated lethal concentration of entomopathogenicity test against RPW at LC_50_ and LC_90_.

Treatments	Estimated LC/EC Value		90% Confidence Level	
			Lower	Upper
Emulsion formulated conidia	LC_50_	1.910 × 10^5^	3.095 × 10^4^	6.092 × 10^5^
	LC_90_	3.893 × 10^6^	3.893 × 10^6^	5.860 × 10^7^
Non-formulated conidia	LC_50_	7.671 × 10^5^	2.049 × 10^5^	2.830 × 10^6^
	LC_90_	2.638 × 10^8^	4.717 × 10^7^	4.301 × 10^9^

**Table 3:** Indirect contact bioassay using emulsion formulated and non-formulated conidia at different concentrations.

Indirect Contact Bioassay				
Treatment	95% Confidence Level			
	LT_50_ (Days)	Lower Bound	Upper Bound	R^2^
Oil-emulsion formulated conidia				
1 × 10^3^ conidia mL^−1^	50.679 ^a^	2.083	13.155	0.946
1 × 10^5^ conidia mL^−1^	21.729 ^b^	12.321	23.394	0.946
1 × 10^7^ conidia mL^−1^	16.104 ^b^	18.035	29.108	0.924
1 × 10^9^ conidia mL^−1^	12.475 ^c^	26.130	37.203	0.961
Non-formulated conidia suspension				
1 × 10^3^ conidia mL^−1^	94.518 ^a^	−1.1965	9.822	0.921
1 × 10^5^ conidia mL^−1^	105.686 ^a^	−1.251	12.203	0.861
1 × 10^7^ conidia mL^−1^	61.387 ^a^	1.130	13.870	0.903
1 × 10^9^ conidia mL^−1^	57.228 ^a^	2.797	14.243	0.906
Positive controls				
Cypermethrin (pesticides)	6.120 ^d^	63.909	75.116	0.935
Ory-X (mycoinsecticides)	0	0	0	0
Negative control				
0.05% Tween 80	0	0	0	0
Emulsion blank				

Note: According to the Student-Newman-Keuls post-hoc test, the LT_50_ values followed by different letters are significantly different at *p* = 0.05 (n = 30). ‘0’ represents zero mortality.

## Data Availability

Not applicable.
